# Stable expression plasmids for *Streptomyces* based on a toxin-antitoxin system

**DOI:** 10.1186/1475-2859-12-39

**Published:** 2013-04-25

**Authors:** Laura Sevillano, Margarita Díaz, Ramón I Santamaría

**Affiliations:** 1 Instituto de Biología Funcional y Genómica. Consejo Superior de Investigaciones Científicas/Universidad de Salamanca, C/ Zacarías González nº2, Salamanca 37007, Spain

**Keywords:** *Streptomyces*, Toxin-antitoxin, Recombinant protein, Plasmid stabilization

## Abstract

**Background:**

Bacteria included in the genus *Streptomyces* exhibit several attractive characteristics that make them adequate hosts for the heterologous expression of proteins. One of them is that some of its species have a high secretion capacity and hence the protein of interest could be released to the culture supernatant, facilitating downstream processing. To date, all the expression vectors described for these bacteria contain antibiotic resistance genes as selection markers. However, the use of antibiotics to produce proteins at industrial level is currently becoming more restricted owing to the possibility of contamination of the final product. In this report, we describe the use of the *S. lividans yefM/yoeBsl* toxin-antitoxin system to develop a stable plasmid expression system.

**Results:**

In order to use the *yefM/yoeBsl* system to stabilize expression plasmids in *Streptomyces*, a *S. lividans* mutant strain that contained only the toxin gene (*yoeBsl*) in its genome and the antitoxin gene (*yefMsl*) located in a temperature-sensitive plasmid was constructed and used as host. This strain was transformed with an expression plasmid harbouring both the antitoxin gene and the gene encoding the protein of interest. Thus, after elimination of the temperature-sensitive plasmid, only cells with the expression plasmid were able to survive. On using this system, two proteins - an α-amylase from *S. griseus* and a xylanase from *S. halstedii -* were overproduced without the addition of antibiotic to the culture medium. The production of both proteins was high, even after long incubations (8 days), and after serial subcultures, confirming the stability of the plasmids without antibiotic selection.

**Conclusions:**

This is the first report that describes the use of a toxin-antitoxin system to maintain high -copy plasmids in *Streptomyces*. This finding could be a valuable tool for using *Streptomyces* as a host to produce proteins at the industrial and pharmaceutical levels without the use of antibiotics in the production step.

## Background

The production of high levels of proteins for different purposes (scientific, therapeutic, diagnostic, environmental, agricultural, etc.) is one of the main goals of biotechnology and the choice of a suitable expression system is a crucial step in this process. Owing to its fast proliferation, high expression levels and short fermentation time, *Escherichia coli* is the host bacterium most commonly used for the large-scale production of recombinant proteins [1]. However, *E. coli* is not always suitable for the production of active proteins on account of problems of insolubility, cytotoxicity, inefficient translation or the inability to carry out post-translational modifications [2]. In order to overcome these problems, other prokaryotic and eukaryotic hosts have been used. *Bacillus* spp and *Lactococcus* are Gram-positive bacteria that are often used to secrete proteins into the culture medium [3-8]. Eukaryotic cells, such as yeast cells, insect cells or immortalized cell lines, are mainly used to produce active proteins with post-translational modifications [9,10].

*Streptomyces* is a promising bacterial expression system that has been used to produce high levels of several proteins [1,11]. Streptomycetes are aerobic, filamentous Gram-positive soil bacteria that secrete a wide range of extracellular enzymes to degrade a broad variety of substrates in order to survive.

As a host, *Streptomyces* has the following advantages over other systems: 1) the formation of inclusion bodies has not been described in the literature; 2) it is a well-suited host for the expression of very GC-rich genes without codon adaptation [12]; 3) it has high protein secretion efficiency, which makes it feasible to obtain the protein of interest in the culture supernatant, facilitating the protein folding and downstream procedures of extraction and purification [11]; 4) the species used to express proteins (see below) display a relatively low level of endogenous extracellular proteolytic activity in comparison with other hosts such as *Bacillus*, where deletion of up to seven extracellular proteases had to be done to improve protein production [13,14].

Among *Streptomyces*, *S. lividans* is the preferred host for producing recombinant proteins because it is genetically well characterized, it has low proteolytic activity and it lacks the restriction systems present in other *Streptomyces* species such as *S. coelicolor.* Accordingly, *S. lividans* can be directly transformed with *E. coli*-derived shuttle vectors. In fact, it has been used to produce a large number of proteins of both prokaryotic and eukaryotic origin. Most of them are summarized in two reviews [1,11].

Our group has developed an expression system for *Streptomyces* based on the use of strong promoter such as the *xysAp* promoter from *S. halstedii*[15] regulated by different carbon sources. The system has proved useful for the expression of genes from different *Streptomyces* species, from *Aspergillus nidulans* and from *Thermus thermophilus* ([12,16]). This system uses multi-copy mono-functional (*Streptomyces*) or bi-functional (*E. coli* and *Streptomyces*) plasmids that are selected with antibiotic resistance genes (mainly thiostrepton or neomycin). However, the use of antibiotics at large-scale entails several problems associated with the potential risk of contamination of the product, apart from its high cost. Moreover, the use of antibiotics in intensive culture conditions is not efficient owing to dilution or antibiotic inactivation [17,18]. This has made it necessary to develop alternative methods to stabilize plasmid-bearing cultures without adding antibiotics.

One alternative strategy is the use of toxin-antitoxin (TA) systems that involve host death upon plasmid loss. TA systems are widespread among the plasmids and genomes of bacteria and archaea and play important roles in responses to environmental stress as well as in the stability of mobile genetic material [19]. Class II TA systems consist of a biologically active protein molecule (toxin) and the corresponding inhibitor protein (antitoxin). The efficiency of these TA systems depends on the different lifespan of both proteins; while toxins are highly resistant to proteases, antitoxins have a very short half-life owing to their high susceptibility to protease activity. Toxin activity places cells in a dormant state, leading to cell death during prolonged exposure. TA systems have been used for the positive selection of transformants in *E. coli*[17,18,20]. Initially, the sequence encoding the full operon was included in the expression plasmid and in plasmid-free cells the antitoxin was degraded, leaving the toxin free to exert its effect and causing cell death [21]. However, this system can only delay the takeover of the culture by plasmid-free cells because the dilution of the toxin, as a consequence of bacterial growth, allows plasmid-free cells to survive. To surmount these problems, other systems have been developed based on the separation of the two components of the operon [17,18]. In these separate-component-stabilization (SCS) systems, the antitoxin is localized in the plasmid and the toxin is integrated in the bacterial chromosome. Thus, when the plasmid is present in the cell, the antitoxin counteracts the effect of the toxin, whereas upon plasmid loss the toxin causes cell death.

Some of these systems based on the separation of the two components of the operon are available commercially for *E. coli* (StabyCloning™ and StabyExpress™, Delphi Genetics SA). In these, the toxin gene (*ccdB*) is integrated into the host chromosome and the antitoxin gene (*ccdA*) is inserted into an expression plasmid, and hence loss of the expression plasmid leads to cell death. They have been shown to be effective for plasmid stabilization without the use of antibiotics during the production process [17,18,20].

By means of bioinformatics tools, up to 24 putative TA systems have been identified in the *S. coelicolor* genome (TADB) [22]. Our group has recently characterized the first toxin/antitoxin system from *Streptomyces* experimentally [23]. This system, from *S. lividans,* was named *yefM/yoeBsl* and is identical to the system encoded by *SCO2235/SCO2236* from *S. coelicolor* (*yefM/yoeBsc*). In this system, the toxin (YoeBsl) acts by inhibiting translation initiation due to its mRNA interferase activity [23].

Here, we describe a new strategy for the stabilization of expression plasmids in *Streptomyces* based on the use of the *yefM/yoeBsl* system as a selection marker. In a first step, we constructed the *S. lividans* host strain that contained only the toxin gene (*yoeBsl*) in the chromosome, while the antitoxin gene (*yefMsl*) was located in a temperature-sensitive plasmid. This strain was used as a host to produce proteins from an expression plasmid that contained both the antitoxin gene and the gene of the protein of interest. Thus, after removal of the temperature-sensitive plasmid containing the antitoxin gene, the loss of the expression plasmid led to cell death. This system was used to produce two proteins without the use of antibiotics during the production process. The production of both proteins remained high even in long-term and serial cultures. These results confirm the efficiency of the selection method as well as the stability of the expression plasmids.

## Results and discussion

### Strategy

**Figure 1:**
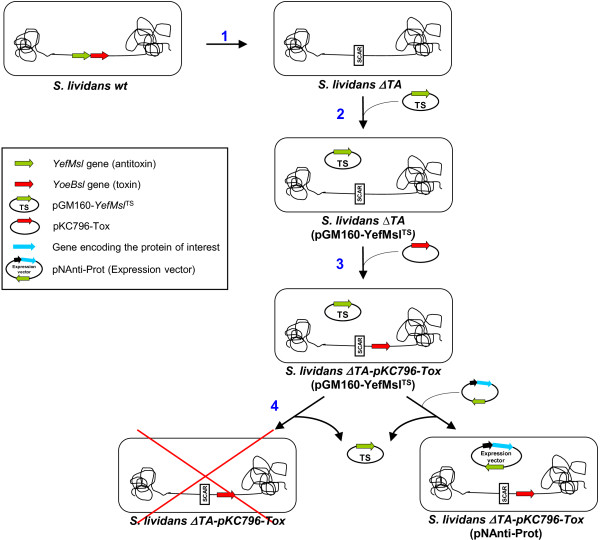
**Separate component-stabilization system in *Streptomyces *strategy. 1**. *S. lividans ΔTA* obtention. **2**. Transformation with the temperature-sensitive plasmid carrying the antitoxin (pGM160-YefMsl^TS^). **3**. Integration of the *yoeBsl* gene into the chromosome of the bacteria with plasmid pKC796-Tox. **4**. Transformation with the expression plasmid (pNAnti-Prot) and removal of the temperature-sensitive plasmid. When the expression plasmid was lost toxin production produced cell death.

In the present work we developed a *Streptomyces* separate-component-stabilization system with the *yefM/yoeBsl* TA genes from *S. lividans*[23]. The strategy followed during the work is summarized in Figure 1. In an initial step, we deleted the complete *yefM/yoeBsl* operon from the genome to obtain the *S. lividans ΔTA* strain (Figure 1 step 1); the integration of *yoeBsl* into the chromosome of the *ΔTA* strain was lethal [23]. Therefore, before integration of the toxin we needed to transform this strain with an easily removable plasmid containing the antitoxin. Thus, the *S. lividans-ΔTA* strain was transformed with a temperature-sensitive plasmid (pGM160 derivate [24]) containing the antitoxin gene (Figure 1 step 2). Then, the toxin gene was integrated in the genome with the integrative plasmid pKC796-Tox [23] (Figure 1 step 3). This was the host strain used to express proteins from plasmids selected with this system (Figure 1 step 4).

### Host strain construction

**Figure 2:**
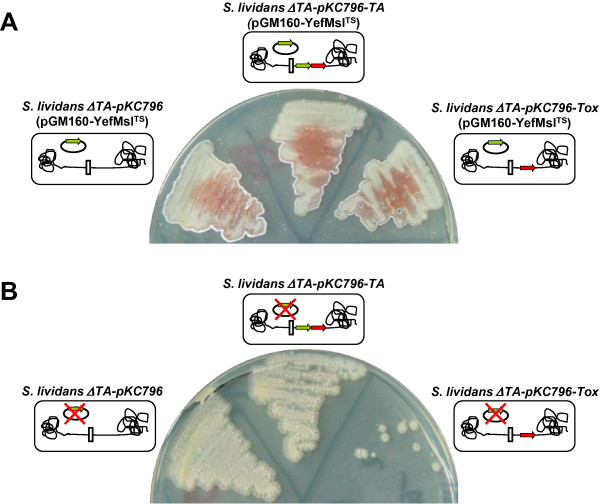
**Temperature effect in the growth of the different Strains.** Growth of the host strain (*S. lividans ΔTA-pKC796-Tox*(pGM160*-*YefMsl^TS^)) at 28°C (**A**) and at 37°C (**B**). Loss of the temperature-sensitive plasmid pGM160-YefMsl^TS^ originated a lethal strain. Strains *S. lividans ΔTA-pKC796(*pGM160*-*YefMsl^TS^) and *S. lividans ΔTA-pKC796-TA(*pGM160*-*YefMsl^TS^) were used as controls.

*S. lividans ΔTA* was obtained by means of REDIRECT technology, as previously described [23]. *S. lividans ΔTA* protoplasts were transformed with the multicopy temperature-sensitive thiostrepton resistance pGM160-YefMsl^ts^ plasmid [23], which expresses YefMsl (antitoxin) under the control of the strong *Streptomyces xysAp* promoter [15,25]. The strain thus obtained was designated *S. lividans ΔTA*(pGM160-YefMsl^ts^) (Figure 1). Then, the *yoeBsl* (toxin) gene was integrated into the genome of this strain with the pKC796-Tox plasmid [23] to yield *ΔTA-pKC796-Tox*(pGM160-YefMsl^ts^). *S. lividans ΔTA*(pGM160-YefMsl^ts^) protoplasts were also transformed with the empty integrative pKC796 plasmid, generating strain *ΔTA-pKC796*(pGM160-YefMsl^ts^), and with the plasmid containing the complete toxin-antitoxin operon, pKC796-TA, generating strain *ΔTA-pKC796-TA*(pGM160-YefMsl^ts^), which were used as controls. Viability studies of these strains under different temperature conditions (28 and 37°C) demonstrated that the *S. lividans ΔTA* strain with the toxin gene integrated in the genome (*ΔTA-pKC796-Tox*(pGM160-YefMsl^ts^)) was only viable at 28°C. It was observed that, when this strain was spread onto R2YE medium and incubated at 37°C the cells died because the temperature-sensitive pGM160-YefMsl^ts^ plasmid had been lost (Figure 2). However, a few surviving colonies were observed but these were not able to grow when they were reinoculated onto patches on R2YE medium, suggesting an accumulative effect of the toxin (data not shown). This strain was therefore suitable for hosting the expression plasmid, with the antitoxin gene as well as the gene encoding the protein of interest. No toxic effect was observed when plasmid pGM160-YefMsl^ts^ was lost in the control strains *ΔTA-pKC796*(pGM160-YefMsl^ts^) and *ΔTA-pKC796-TA*(pGM160-YefMsl^ts^) when incubated at 37°C (Figure 2).

### Effectiveness of the *yefM/yoeBsl* system as a plasmid stabilization method

The efficiency of the *yefM/yoeBsl* system in the maintenance of expression plasmids without antibiotic selection was studied using the *S. lividans ΔTA-pKC796-Tox*(pGM160-YefMsl^ts^) strain as host. The production of two proteins - the Amy α-amylase from *S. griseus* IMRU3570 and the Xys1 xylanase from *S. halstedii* JM8 - was studied in this strain and compared with the one obtained with the *wt-pKC796* and *ΔTA-pKC796*(pGM160-YefMsl^ts^) strains, used as controls.

Plasmid pN702Gem3-Anti, a multicopy *Streptomyces* plasmid that harbours the *yefMsl* gene under the control of the xylanase promoter *xysAp* (see Methods), was used to make the expression plasmids by cloning the genes encoding the Amy α-amylase or the Xys1 xylanase under the control of the *pstSp* promoter (see Methods).

**Figure 3:**
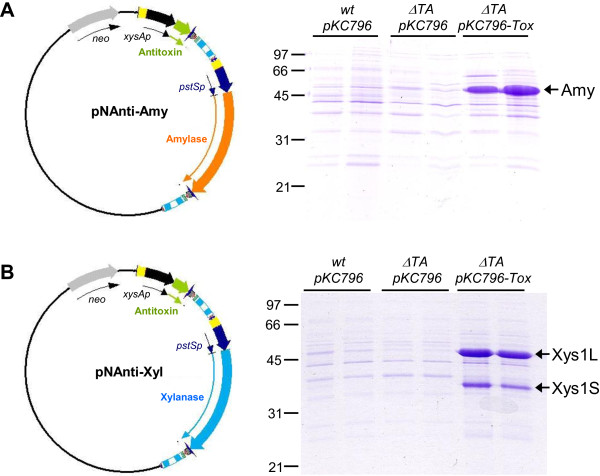
**Enzyme production by the different strains.** Amylase (**A**) and Xylanase (**B**) production by *S. lividans wt-pKC796*, *S. lividans ΔTA-pKC796* and *S. lividans ΔTA-pKC796-Tox* transformed with pNAnti-Amy (**A**) and pNAnti-Xyl (**B**) after 4 days of culture in YES medium supplemented with 3% xylose. 10 μL of supernatant was loaded in each track. On the left, a diagram of the expression plasmids is shown.

Two plasmids, pNAnti-Amy and pNAnti-Xyl, were generated (see methods and Figure 3) and were used to transform the *S. lividans ΔTA-pKC796-Tox*(pGM160-YefMsl^ts^) strain and the *S. lividans wt-pKC796* and *S. lividans ΔTA-pKC796*(pGM160-YefMsl^ts^) control strains.

After removal of the temperature-sensitive plasmid (pGM160-YefMsl^ts^) by incubation at 37°C (see Methods), the transformants of *S. lividans ΔTA-pKC796-Tox*(pNAnti-Amy), *S. lividans ΔTA-pKC796-Tox*(pNAnti-Xyl) and the corresponding control strains obtained were grown without antibiotics at 28°C in liquid YES medium supplemented with 3% xylose (an inducer for *xysA* and *pstS* promoters). The amount of amylase and xylanase in the supernatants was analyzed by SDS-PAGE (Figure 3).

After 4 days of culture, strain *ΔTA-pKC796-Tox* produced high levels of amylase (pNAnti-Amy) and xylanase (pNAnti-Xyl) (Figure 3A and B). However, no protein production was observed in the control strains (*wt-pKC796* and *ΔTA-pKC796*) transformed with the same plasmids. These results indicated that the absence of antibiotic in the culture medium was responsible for the loss of the expression plasmids (pNAnti-Amy or pNAnti-Xyl) in the control strains that did not harbour the toxin in their genomes. On the contrary, the strain with the toxin gene integrated in the genome needed the presence of the expression plasmid (in reality, the antitoxin gene present in it) to live. Thus, even without antibiotics, this strain was able to maintain the plasmids and, as consequence, produced high levels of proteins.

These results show that the separate-component-stabilization strategy described in this work allows the over-production of proteins without the addition of antibiotics during the production step.

### Plasmid stability

Plasmid stability without antibiotic selective pressure was evaluated by analyzing the variations in protein production under three different conditions: 1) long-term cultures, 2) after freezing the mycelium and generating new colonies for use as inoculum and 3) after performing serial liquid cultures.

**Figure 4:**
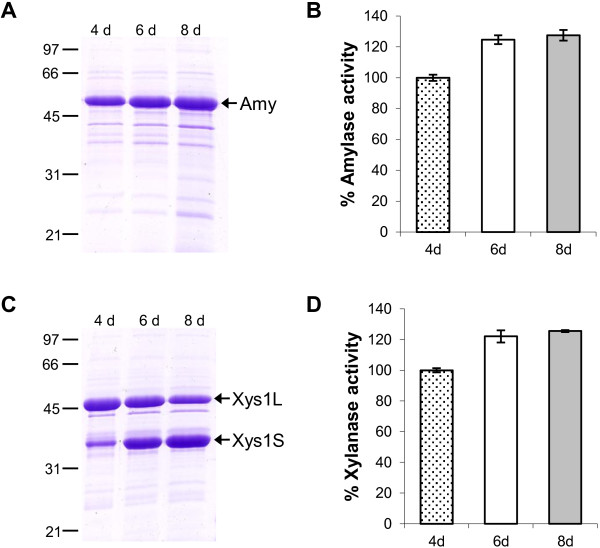
**Protein production in long time cultures without selection.** Amylase (**A**) and Xylanase (**C**) production by *S. lividans ΔTA-pKC796-Tox* transformed with pNAnti-Amy (**A**) and pNAnti-Xyl (**C**) after 4, 6 and 8 days of culture in YES medium supplemented with 3% xylose. 10 μL of supernatant was loaded in each track. **B** and **D** show the percentage of amylase (**B**) and xylanase (**D**) activity of the supernatants. The histogram bars are the mean of four experiments.

The amount of protein produced in long-term cultures was evaluated by SDS-PAGE and by assaying the enzymatic activities in the supernatants of *S. lividans ΔTA-pKC796-Tox* transformed with pNAnti-Amy or with pNAnti-Xyl. High amounts of both proteins were observed even after 8 days of culture (Figure 4), suggesting that the *yefM/yoeBsl* system allows stable maintenance of the plasmids that express the amylase or the xylanase in long-term cultures.

**Figure 5:**
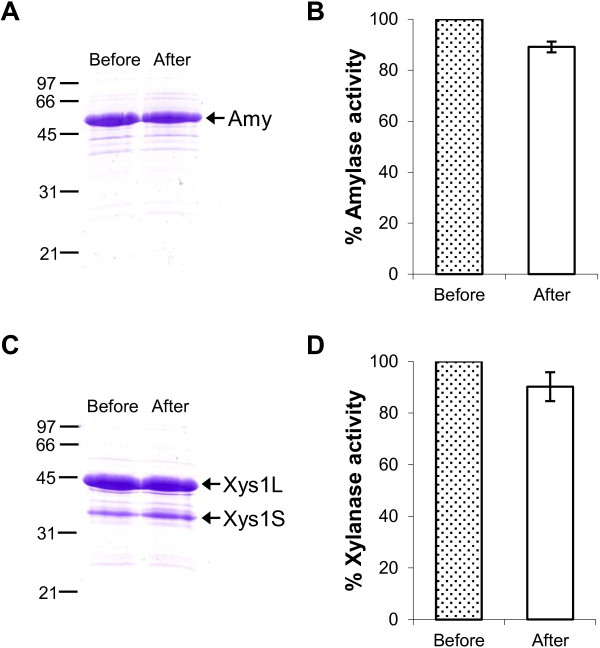
**Protein production after a freezing step of the mycelium.** Amylase (**A**) and Xylanase (**C**) production by *S. lividans ΔTA-pKC796-Tox* transformed with pNAnti-Amy (**A**) and pNAnti-Xyl (**C**) before and after freezing the mycelium. 10 μL of 4-day supernatants was loaded in each track. **B** and **D** show the percentages of amylase (**B**) and xylanase (**D**) activity of the supernatants. The histogram bars are the mean of four experiments.

The stability of these plasmids after freezing the mycelium was studied by freezing two-day-old liquid cultures of *S. lividans ΔTA-pKC796-Tox* transformed with pNAnti-Amy or with pNAnti-Xyl at −80°C in 20% glycerol. Frozen cells were streaked out for single colonies on R2YE medium without antibiotics, which were then reinoculated on patches on new R2YE plates without antibiotics and used to inoculate liquid YES medium with 3% xylose. After 4 days of culture at 28°C, amylase and xylanase production was analyzed by SDS-PAGE and measuring the enzymatic activity of culture supernatants. The yield obtained was compared with that achieved with the original 4-day-culture transformants (Figure 5). Over 90% of amylase and xylanase activity persisted when the cultures were set up from frozen mycelium (Figure 5B and D).

**Figure 6:**
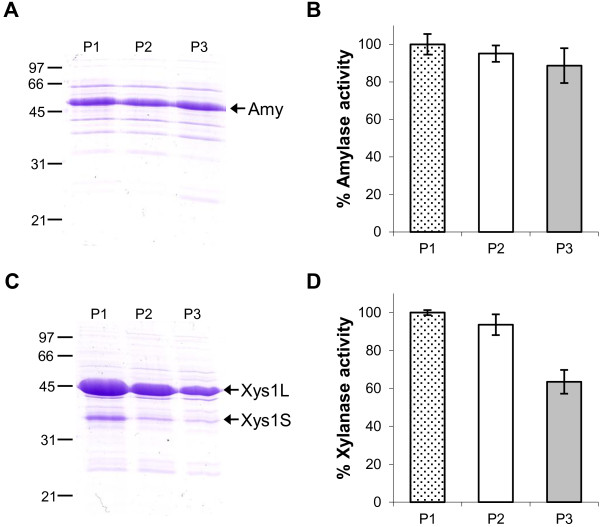
**Protein production after serial subcultures.** Amylase (**A**) and Xylanase (**C**) production by *S. lividans ΔTA-pKC796-Tox* transformed with pNAnti-Amy (**A**) and pNAnti-Xyl (**C**) after three serial subcultures every two days (P1, P2, and P3) in fresh YES medium supplemented with 3% xylose. 10 μL of 4-day supernatants was loaded in each track. **B** and **D** show the percentage of amylase (**B**) and xylanase (**D**) activity of the supernatants. The histogram bars are the mean of four experiments.

Finally, plasmid stability in the absence of antibiotic selection was assessed by serial 100-fold dilutions of cultures in fresh YES medium every 48 hours over 6 days. The amylase and xylanase activities of each subculture were analyzed in the supernatants and compared with the enzymatic activity obtained in the original culture (Figure 6). The amylase activity obtained after three rounds of subculture was 90% of the original culture activity (Figure 6B). Xylanase production was also high throughout the experiment but a loss of xylanase activity was observed in the third subculture, where it was 70% of that obtained in the original culture (Figure 6D).

These data show that the toxicity of the product of the integrated toxin gene (*yoeBsl*) in the genome of the host strain (*S. lividans ΔTA-pKC796-Tox*(pNAnti-“prot”)) is able to allow stable maintenance of the multicopy expression plasmids carrying the antitoxin gene (*yefMsl*) and the gene encoding the protein of interest (pNAnti-“prot”).

## Conclusions

In this work, we report a new method that allows the production of high levels of proteins in *Streptomyces* without the use of antibiotics during the production step. This new system is based on the separation of the toxin gene (localized in the genome) and antitoxin gene (localized in the expression plasmid) of the *yefM/yoeBsl* operon from *S. lividans*.

The system was found to be useful for the production of high yields of two proteins and could be a powerful tool for producing other proteins of interest in *Streptomyces* without the drawbacks associated with the use of antibiotics in the production step. This work constitutes the first application of a toxin-antitoxin system to select and stabilize plasmids in *Streptomyces*.

## Methods

### Bacterial strains and growth conditions

The *E. coli* DH5α strain [26] was used for the cloning and isolation of plasmids. All strains were grown in Luria-Bertani (LB) liquid broth or on LB agar. All manipulations in *E. coli* were performed following standard procedures [26].

Protoplasts of *S. lividans* 1326 and *S. lividans ΔTA* (Δ*yefM/yoeBsl*) were grown on solid R2YE medium after transformation [27]. MSA medium was used for *Streptomyces* sporulation [27], and liquid YES medium (1% yeast extract, 10.3% sucrose pH 7.2, 5 mM MgCl_2_), supplemented with 0.5% glucose and 0.5% glycine for collecting cells to generate protoplasts, and YES medium supplemented with 3% xylose for protein expression were used. Liquid cultures were carried out in baffled flasks at 28°C and 200 rpm. All manipulations in *Streptomyces* were done as indicated by Kieser [27].

### Transformation of host strain and colony selection

Protoplast of *S. lividans ΔTA-pKC796-Tox*(pGM160-YefMsl) and the control strains, *S. lividans wt-pKC796* and *S. lividans ΔTA-pKC796*(pGM160-YefMsl) were transformed with the expression plasmids. After transformation, protoplasts were regenerated o/n at 28°C, then overlayed with 50 μg/mL neomycin to select the cells transformed with the expression plasmids and transferred to 37°C for 3 days to eliminate the temperature-sensitive plasmid (pGM160-YefMsl). The loss of the temperature-sensitive plasmid (pGM160-YefMsl) in these transformants colonies was checked by replica-plating the clones onto R2YE plates with 10 μg/mL thiostrepton. Finally, the clones were reinoculated on patches on R2YE plates without antibiotics and incubated at 28°C.

### Plasmid constructions

#### pN702Gem3-Anti

*yefMsl* was amplified by PCR from *S. lividans* 1326 genomic DNA using primers LS-005 and LS-022 (Table 1). The resulting fragment was digested with NdeI and XhoI and ligated into plasmid pXHis1 [28] (Table 2) digested with the same enzymes to obtain plasmid pXHis1-Anti, which was used as an intermediate plasmid. Plasmid pN702Gem3-Anti was obtained by digesting pXHis1-Anti with BglII, purifying the corresponding *yefMsl* band and ligating it into pN702Gem3 [29] digested with the same enzyme. In this plasmid, the antitoxin (*yefMsl*) gene is regulated by the *xysA* promoter [15,25].

**Table 1:** Oligonucleotides used

**Name**	**Sequence 5′-3′**	**Use**
**LS-005**	TTTTTTCATATGTCCATCACCGCCAGCGAAG	Forward oligonucleotide to amplify *yefMsl*. The sequence recognized by NdeI is underlined.
**LS-022**	TTTTTTCTCGAGCGCCCGCTCCGCGTCCGGG	Reverse oligonucleotide to amplify *yefMsl*. The sequence recognized by XhoI is underlined.
**MRG-11**	TTTTTTCATATGGCCCGCAGACTCCGCACC	Forward oligonucleotide to amplify the amylase gene *(amy)* from *S. griseus*. The sequence recognized by NdeI is underlined.
**MRG-12**	TTTTTTCTCGAGGCCGCGCCAGGTGTCGTTGAG	Reverse oligonucleotide to amplify the amylase gene *(amy)* from *S. griseus*. The sequence recognized by XhoI is underlined.

**Table 2:** Plasmids used

**Plasmid**	**Characteristics**	**Reference**
**pGM160**	*E.coli*/*Streptomyces* shuttle vector. Thiostrepton and gentamicin resistance.	[24]
**pGM160-YefMsl**	pGM160 derivative. The *xysA* promoter from *S. halstedii* controls *yefMsl* expression.	[23]
**pKC796**	*E.coli*/*Streptomyces* shuttle vector. Apramycin resistance. Integrative plasmid.	[30]
**pKC796-Tox**	pKC796 derivative. The *xysA* promoter from *S. halstedii* controls toxin expression.	[23]
**pKC796-TA**	pKC796 derivative. The *xysA* promoter from *S. halstedii* controls TA expression.	[23]
**pXHis1**	pBluescript SK derivative. Ampicillin resistance. The *xysA* promoter from *S. halstedii* controls *xys1Δ* expression.	[28]
**pXHis1-Anti**	pXHis1 derivative. The *xysA* promoter from *S. halstedii* controls antitoxin expression.	This work
**pN702Gem3**	*E.coli*/*Streptomyces* shuttle vector. Neomycin resistance. High-copy number.	[29]
**pN702Gem3-Anti**	pN702Gem3 derivative. The *xysA* promoter from *S. halstedii* controls antitoxin expression.	This work
**pNUF5**	pN702Gem3 derivative. The *pstS* promoter from *S. lividans* controls xylanase expression.	[31]
**pNUF-Amy**	pNUF5 derivative. The *pstS* promoter from *S. lividans* controls amylase expression.	This work
**pNAnti-Xyl**	pN702Gem3-Anti derivative. The *xysA* promoter from *S. halstedii* controls antitoxin expression and the *pstS* promoter from *S. lividans* controls xylanase expression.	This work
**pNAnti-Amy**	pN702Gem3-Anti derivative. The *xysA* promoter from *S. halstedii* controls antitoxin expression and the *pstS* promoter from *S. lividans* controls amylase expression.	This work

#### pNAnti-Xyl

This plasmid contained the ORF of the xylanase *xysA* gene from *S. halstedii* under the control of the *pstS* promoter and the ORF coding for the antitoxin (YefMsl) under the control of the *xysA* promoter. The plasmid was originated from the pNUF5 plasmid [31], which contains the xylanase gene regulated by *pstSp*; pNUF5 was digested with BglII and BamHI, and the *xysA* gene band was ligated into pN702Gem3-Anti digested with BamHI.

#### pNUF-Amy

The amylase gene *(amy)* from *S. griseus* IMRU 3570 [32] was amplified by PCR using primers MRG-11 and MRG-12 (Table 1). The resulting fragment was digested with NdeI and XhoI and, in different steps, was introduced into pNUF5 downstream the *pstS* promoter.

#### pNAnti-Amy

This plasmid contains the ORF of the amylase gene from *S. griseus* under the control of the *pstS* promoter and the ORF coding for the antitoxin (YefMsl) under the control of the *xysA* promoter. To construct the plasmid, plasmid pNUF-Amy, which contains the amylase gene regulated by *pstSp*, was digested with BglII and BamHI and the corresponding band was ligated into pN702Gem3-Anti digested with BamHI.

### Sequence analyses

All constructions were sequenced in both strands using a Perkin Elmer ABI Prism 377 DNA sequencer. *In silico* plasmids were obtained with the Gene Construction Kit software (GCK, Textco).

### Protein analyses

Protein profiles were analyzed by denaturing polyacrylamide gel electrophoresis (SDS-PAGE) in a MiniProtean II system (Bio-Rad). Proteins were detected by 0.5% Coomassie brilliant blue R staining and low-molecular weight standards from Bio-Rad were used as markers.

### Xylanase and amylase activities assays

Xylanase and amylase activities were measured with the dinitrosalicylic acid (DNS) method, using xylose or maltose as standards respectively [33]. One unit of xylanase or amylase activity was defined as the amount of enzyme required to release 1 μmol of reducing sugars in one minute (expressed as xylose or maltose equivalents respectively). All data shown are means of at least three different experiments.

## Competing interests

The authors declare that they do not have competing interests.

## Authors’ contributions

LS performed most of the experimental work and wrote the manuscript; MD and RS designed the experiments, directed the work, and wrote the manuscript. All authors have read and approved the final version of the manuscript.
